# Clinical outcomes of full arthroscopic combined latissimus dorsi and teres major transfers for irreparable subscapularis tears

**DOI:** 10.1002/jeo2.70226

**Published:** 2025-04-03

**Authors:** Bo Taek Kim, Luis Alfredo Miranda, Chang Hee Baek, Jung Gon Kim, Luis Leoncio Temoche Diaz, Gyu Rim Baek, Jean Kany

**Affiliations:** ^1^ Department of Orthopaedic Surgery Yeosu Baek Hospital Jeollanam‐do Republic of Korea; ^2^ Department of Orthopaedic Surgery Hospital Cima Hermosillo Sonora México; ^3^ Hospital Nacional Cayetano Heredia Lima Perú; ^4^ Catholic Kwandong University School of Medicine Gangwon‐do Republic of Korea; ^5^ Clinique de l'Union, Ramsay Santé Saint Jean France

**Keywords:** combined latissimus dorsi teres major, irreparable rotator cuff tear, irreparable subscapularis tear, latissimus dorsi, tendon transfer, teres major

## Abstract

**Purpose:**

Managing irreparable subscapularis tears poses a challenge. Traditionally, pectoralis major transfer has been the gold standard, but alternative methods including anterior latissimus dorsi (LD) and teres major (TM) tendon transfers have shown promise. This study evaluates the clinical outcome of full arthroscopic combined LDTM transfers for irreparable subscapularis tears.

**Methods:**

Patients with irreparable subscapularis tears who underwent full arthroscopic combined LDTM transfers from October 2020 to August 2022 were retrospectively reviewed. The inclusion criteria comprised failure of conservative treatment, irreparable subscapularis tears (Lafosse grade ≥ 4), and no to minimal glenohumeral arthritis (Hamada grade < 3). The exclusion criteria included patients with missing clinical data. Clinical assessments included the visual analogue scale (VAS), the Constant score, the Subjective Shoulder Value (SSV), active range of motion (ROM), and internal rotation strength. A total of 11 patients met the inclusion criteria and were included in this study.

**Results:**

Out of 14 patients, 11 met the inclusion criteria, with a mean age of 65.9 ± 6.0 years and a follow‐up duration of 25.9 ± 6.2 months. Postoperative results demonstrated significant pain relief, with the VAS score improving from 8.1 ± 0.9 to 2.1 ± 2.1 (*p* < 0.001). Functional outcomes improved significantly, with the Constant score increasing from 29.9 ± 3.9 to 62.6 ± 15.9 (*P* < 0.001) and the SSV improving from 25.3 ± 8.7 to 66.5 ± 20.1 (*p* < 0.001). ROM significantly increased in forward elevation, abduction, and internal rotation, while internal rotation strength also improved significantly. There were no complications or progression of arthritis observed.

**Conclusion:**

Full arthroscopic combined LDTM transfer demonstrates promising clinical and radiological short‐term outcomes for patients with irreparable subscapularis tears. The procedure resulted in substantial improvements in pain relief and functional outcomes, particularly in internal rotation for both ROM and strength. Importantly, no significant complications or progression of glenohumeral arthritis were observed by the final follow‐up.

**Level of Evidence:**

Level IV, retrospective case series.

**Abbreviations**
ABD: abduction
ACR: anterior capsular reconstruction
ADLIR: Activities of Daily Living Index Rating
ASES: American Shoulder and Elbow Surgeons score
D: distal
ERS: external rotation at the side
FE: forward elevation
IL: inferior lateral
IM: inferior medial
IR: internal rotation
LD: latissimus dorsi
LDTM: latissimus dorsi and teres major
MRI: magnetic resonance imaging
PM: pectoralis major
ROM: range of motion
SL: superolateral
SSV: Shoulder Value
TM: teres major
UCLA: University of California‐Los Angeles score
VAS: visual analogue scale

## INTRODUCTION

Managing irreparable subscapularis tears, particularly in young and active elderly patients, remains a significant clinical challenge [10, 11, 26]. Traditionally, the pectoralis major (PM) transfer has been considered the gold standard for treating these tears. This procedure involves transferring either the sternal head, clavicular head, or both, with the tendon routed either above or below the conjoint tendon [9, 11, 14, 21, 27, 29]. However, alternative tendon transfer methods, such as pectoralis minor or upper trapezius transfers, have also been explored, though they are less supported by extensive studies and lack long‐term follow‐up date [11, 18, 27]. Another treatment option, anterior capsular reconstruction (ACR), has been proposed, but its biomechanical and clinical outcomes have not yet been sufficiently validated for widespread use [25, 28].

More recently, anterior latissimus dorsi (LD) tendon transfer has emerged as a promising option for managing irreparable subscapularis tears [3, 13, 24]. Some studies suggest that LD transfer may offer biomechanical advantages over PM transfer, particularly in improving internal rotation (IR) of the shoulder [7, 12, 21, 25]. The combined LD and teres major (TM) transfer has been proposed as a treatment option for irreparable subscapularis tears to improve the shoulder stability and facilitate more effective humeral head depression [12]. Biomechanical studies support the superiority of combined LDTM transfer for irreparable subscapularis in restoring humeral head positioning and improving IR [4, 5, 6]. Although clinical results of combined LDTM transfer have been promising, they have predominantly been achieved through open surgery [1, 2]. To date, there have been no clinical results published on full arthroscopic combined LDTM transfer for irreparable subscapularis tears.

This study aims to evaluate the efficacy of full arthroscopic combined LDTM transfer for reconstructing irreparable subscapularis tears. We hypothesise that this technique will yield promising clinical results and serve as an alternative to traditional open approaches.

## MATERIALS AND METHODS

This study was approved by the Institutional Review Board (COS‐RGDS‐2025‐01‐005‐KANY‐J). The requirement for informed consent was waived owing to the retrospective design of the study and the lack of additional harm to the patients.

### Patients selection

We retrospectively reviewed the medical records of patients who underwent full arthroscopic combined LDTM tendon transfers for irreparable subscapularis tear between October 2020 and August 2022. Inclusion criteria included persistent shoulder pain and dysfunction that significantly impaired daily activities despite 3–6 months of conservative treatment, irreparable subscapularis tears classified as Lafosse [20] grade ≥ 4, high fatty infiltration in subscapularis with Goutallier [15] grade ≥ 3, intact LD and TM muscle function, and the absence of advanced glenohumeral arthritis, as Hamada [17] stage < 3. Patients with missing clinical assessment data were excluded from the study.

### Surgical procedure

**Figure 1:**
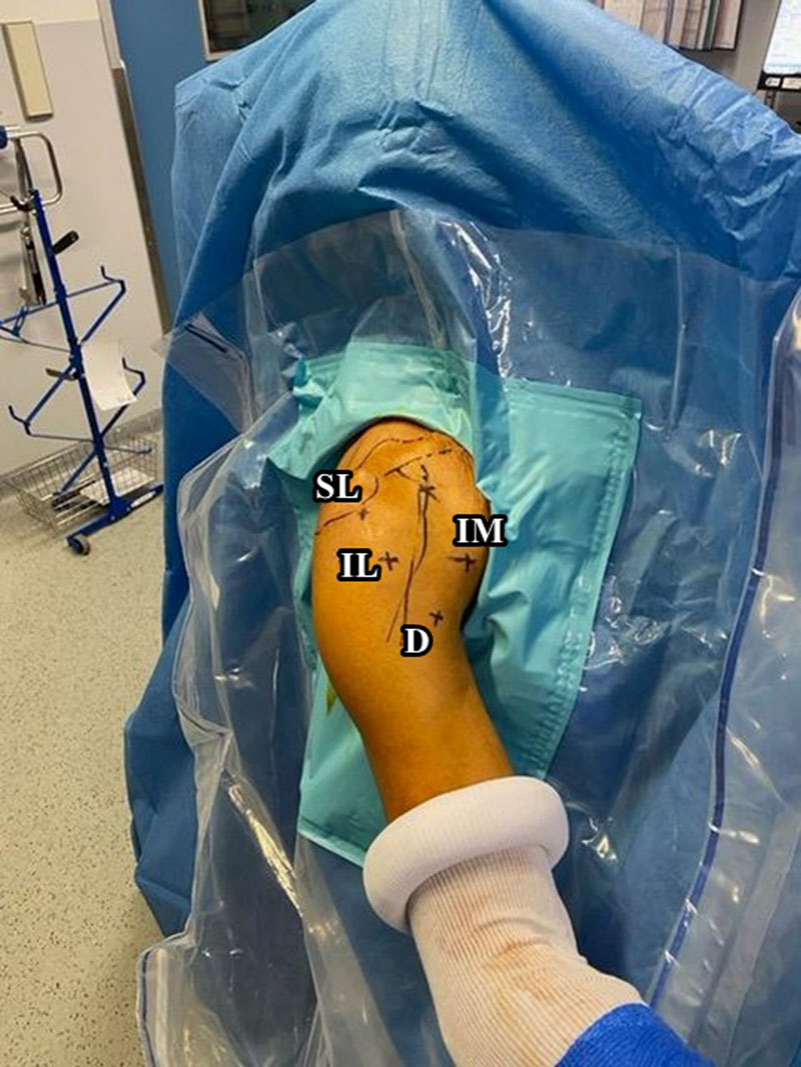
Superolateral (SL) portal near the anterior acromion; Inferior medial (IM) portal along the long head of the biceps tendon, inferior lateral (IL) portal located distal to the SL; distal (D) portal lateral to the lower insertion of the pectoralis major (PM).

All surgeries were performed by a single senior surgeon (J.K) at a single centre. For the full arthroscopic combined LDTM transfer, patients were positioned in the beach chair, and general anaesthesia was administered along with an interscalene nerve block. The arm was secured using a traction device (Spider; Smith & Nephew, Andover, MA, USA) to facilitate optimal surgical access. The entire procedure, including harvesting, preparation, and fixation of the LDTM tendon, was performed arthroscopically. The procedure utilised four key arthroscopic portals: the superolateral (SL) portal near the anterior acromion, the inferior medial (IM) portal along the long head of the biceps tendon, the inferior lateral (IL) portal located distal to the SL, and the distal (D) portal lateral to the lower insertion of the PM (Figure 1). These portals provided excellent visualisation and instrument access.

Full arthroscopic combined LDTM transfer was performed when the subscapularis tendon was confirmed to be completely detached from the lesser tuberosity and irreducible to its original footprint. First, subacromial and subdeltoid debridement was carried out, ensuring preservation of the coracoacromial ligament to prevent superior migration of the humeral head. For the tears in long head of the biceps, either a tenotomy (*n* = 2) or tenodesis (*n* = 2) was performed depending on its arthroscopic aspect. In cases where full‐thickness supraspinatus tendon tears were present and repairable (*n* = 1), these were addressed before proceeding with the combined LDTM transfer.

**Figure 2:**
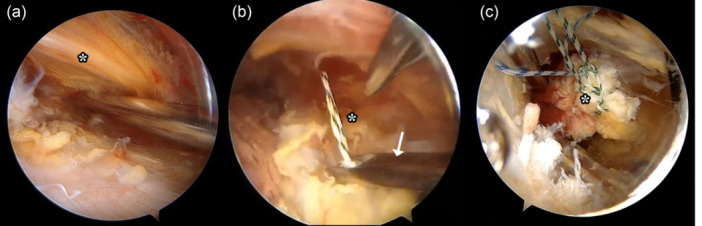
(a) Arthroscopic image of left shoulder from inferior lateral portal view showing radial nerve (white asterisk). (b) #2 Suture was applied to the detached distal portion of the latissimus dorsi and teres major tendon (white asterisk), using self‐retrieving suture passer (white arrow). (c) Prepared latissimus dorsi and teres major tendon (white asterisk).

For the harvesting, preparation, and fixation, IM, IL, and D portals were mainly used. The insertion site for the LD and TM on the humerus was identified, and the distal ends of both muscles were carefully detached using a radiofrequency ablator. During this procedure, special attention was given to protecting the radial nerve, which is typically located 3–4 cm medial to the humeral insertion of the LD (Figure 2a). A triple Krackow suture (#2) was applied to the detached distal portion of the LDTM tendon, using self‐retrieving suture passer (Figure 2b). Following this, the proximal portion of the LDTM was gradually released, and a second Krackow suture was placed in the proximal portion. Once fully released, the ‘three sisters’ vessels and the axillary nerve were identified to ensure the safe separation of the upper LDTM from the residual subscapularis tissue. The LDTM was then carefully separated from surrounding adhesions to enhance excursion and advanced distally, ensuring clearance from the intermuscular septum and radial nerve (Figure 2c).

**Figure 3:**
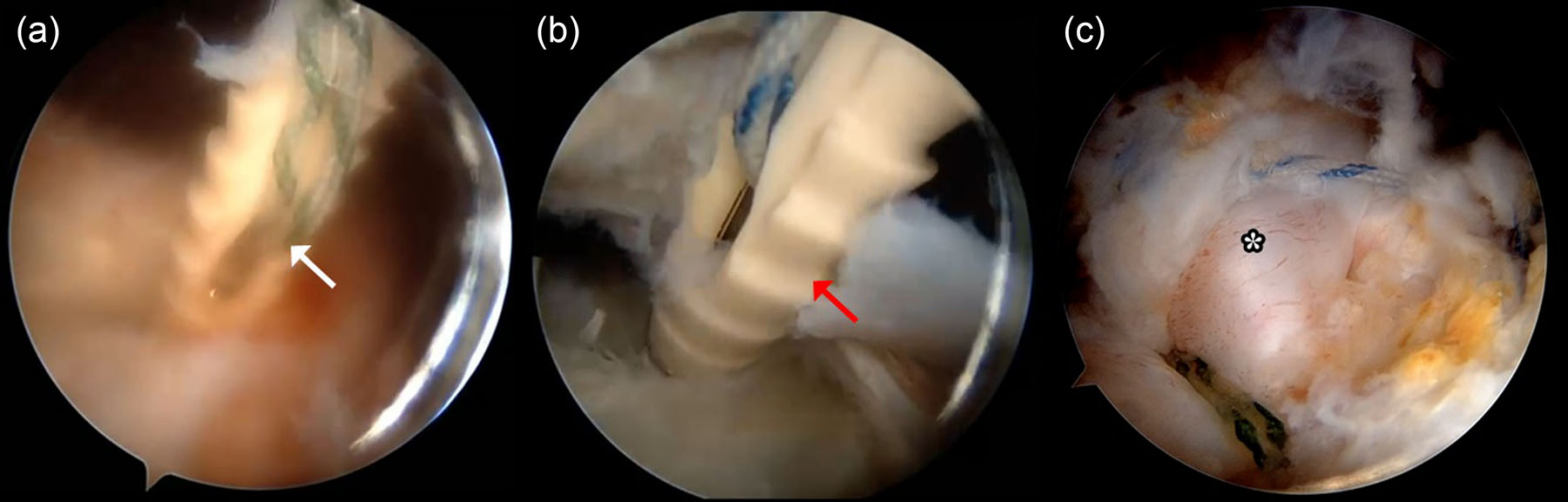
Arthroscopic image of left shoulder from inferior lateral portal view showing (a) first knotless anchor (white arrow) and (b) second knotless anchor (red arrow) fixed to mid‐upper portion of the lessor tuberosity. (c) Arthroscopic image of left shoulder from superior lateral portal view showing final appearance of latissimus dorsi and teres major transfer (white asterisk).

Next, the lesser tuberosity of the humerus was prepared through thorough debridement and decortication to create a suitable attachment site for the tendon transfer. The prepared LDTM was securely anchored using two knotless Quattro® Link Knotless SP anchors (Zimmer Biomet, Warsaw, IN, USA), positioned medially and laterally across the mid‐upper portion of the lesser tuberosity, extending into the bicipital groove (Figure 3). Stability of the fixation was confirmed through intraoperative testing of internal and external shoulder rotation.

### Clinical assessment

All data were collected by both a senior surgeon (J.K) and a junior fellow (L.M). Clinical assessments were obtained preoperatively and at the final follow‐up. Patient demographic data, including age, sex, length of symptoms, and any relevant medical comorbidities, were recorded. Pain intensity was measured using the visual analogue scale (VAS), while shoulder function was evaluated through the Constant score and Subjective Shoulder Value (SSV). Active range of motion (ROM) was assessed for forward elevation (FE), abduction (ABD), and external rotation at the side (ERS) using a standardised goniometer. IR to back was evaluated by correlating the thumb position to vertebral levels on the following scale: 0—Greater trochanter of the femur; 1—Sacrum; 2—Lumbar 5 vertebra; 3—Lumbar 3 vertebra; 4—Lumbar 1 vertebra; 5—Thoracic 12 vertebra; 6—Thoracic 10 vertebra; 7—Thoracic 8 vertebra; 8—Thoracic 6 vertebra. Muscle strength was assessed using the modified British Medical Research Council scale [22]. Preoperative subscapularis function was evaluated through a series of specialised physical tests, including the belly press, bear hug and lift‐off tests [19]. A positive result for the belly press test was defined as the patient being unable to resist pressure applied during the test. For the bear hug test, a positive result was recorded if the patient was unable to maintain their hand position on the opposite shoulder when pressure was applied. Similarly, a positive result for the lift‐off test was defined as the patient being unable to lift their hand off their back or maintain the lifted position [19].

### Radiologic assessment

Preoperative imaging assessments were done by single senior surgeon (J.K.), which involved plain radiographs in anteroposterior, scapular Y, and axillary views, along with magnetic resonance imaging (MRI) to confirm the extent of the subscapularis tear and any associated shoulder pathology. Postoperative radiographs were performed at the final follow‐up, and the integrity of the transferred tendon was assessed using sonographic examination by radiologist. Proximal humeral migration was evaluated using plain radiographs by comparing true anteroposterior views between the preoperative and final follow‐up period.

### Postoperative rehabilitation

The postoperative rehabilitation protocol was divided into multiple phases. For the first 0–4 weeks, the shoulder was immobilised in IR to protect the surgical site. From 4 to 12 weeks, patients advanced to passive and active‐assisted ROM exercises, with active movements allowed, but external rotation limited to 30°. Pool‐based exercises were recommended to facilitate gentle motion and reduce joint stress. From 12 to 24 weeks, external rotation restrictions were lifted, and elastic band exercises were introduced to strengthen the shoulder. Also, during this period, full internal and external rotation exercises were incorporated, alongside strengthening exercises for abduction. By 6 months, patients were allowed to resume unrestricted activities, marking the final phase of rehabilitation.

### Statistical analysis

Paired *t*‐tests and Wilcoxon signed‐rank tests were used to compare preoperative and final clinical parameters for continuous data. For categorical variables, Chi‐square and Fisher's exact tests were applied. All statistical analyses were performed using SPSS for Windows, version 19.0 (IBM, Armonk, NY, USA).

## RESULTS

Following the exclusion of four patients, 11 patients met the inclusion criteria. The mean age of these patients was 65.9 ± 6.0 years (range: 54–73 years), with an average follow‐up duration of 25.9 ± 6.2 months (range: 24–36 months). Preoperative radiography revealed no to minimal glenohumeral arthritis in all patients, with Hamada [17] grades of 1 or 2; none exhibited Grade 3. Preoperative MRI confirmed irreparable subscapularis tears with medial retraction and significant fatty infiltration, showing Goutallier [15] grade 3 fatty infiltration in 36.3% of patients and Grade 4 in 63.6%. Among the 11 patients, nine had concomitant supraspinatus tears, of which only one was deemed repairable; the remaining eight were considered irreparable with Goutallier grade [15] 3 or 4 fatty infilltration. All patients had intact infraspinatus and teres minor. Table 1 summarises the patient demographics and clinical information in detail.

**Table 1:** Demographics.

Variables	Total (*n* = 11)
Sex, male/female, *n* (%)	6 (54.5)/5 (45.4)
Age (year), mean ± SD (range)	65.9 ± 6.0 (54‐73
Follow‐up (month), mean ± SD (range)	25.9 ± 6.2 (24‐36)
Length of symptoms (month)	23.2 ± 32.1 (2‐96)
Dominant arm involvement, *n* (%)	8 (72.7)
RA, *n* (%)	0 (0.0)
DM, *n* (%)	0 (0.0)
Smoking, *n* (%)	0 (0.0)
Belly press, *n* (%)	11 (100.0)
Lift off, *n* (%)	11 (100.0)
Bear hug, *n* (%)	11 (100.0)
Concomitant SSP tear	9 (81.8)
Concomitant SSP repair	1 (9.1)
Prior surgery, a/s rotator cuff repair, *n* (%)	3 (27.2)
Preoperative SSC FI grade, *n* (%)	
Grade 3	4 (36.3)
Grade 4	7 (63.6)

Abbreviations: DM, diabetes mellitus; FI, fatty infiltration; RA, rheumatoid arthritis; SD, standard deviation; SSC, subscapularis; SSP, supraspinatus.

Table 2 summarises the preoperative and postoperative clinical outcomes of the patients. Postoperative outcomes revealed a significant decrease in VAS scores, while functional assessments—measured by the Constant score and the SSV—also showed significant improvement. Significant changes were observed in ROM, including FE, ABD, and IR (*p* < 0.001). IR strength increased from 1.0 ± 0.0 preoperatively to 3.5 ± 0.9 postoperatively (*p* < 0.001). Preoperative humeral head subluxation was identified in four patients, which decreased to three postoperatively. Proximal humeral migration was present in two patients preoperatively and observed in three patients postoperatively. One patient experienced a retear, confirmed through sonographic evaluation; however, as the patient reported a reduction in pain (VAS_pre_ 8 to VAS_final_ 2) and patient reported outcome measures (Constant_pre_ 35 to Constant_final_ 55, SSV_pre_ 25 to SSV_final_ 60), further surgical intervention was not performed. There were no cases of arthritis progression, and no significant complications were observed by the final follow‐up.

**Table 2:** Postoperative clinical outcome.

Variables	Preoperative	Final follow‐up	*p*‐Value
VAS pain score	8.1 ± 0.9	2.1 ± 2.1	<0.001
Constant score	29.9 ± 3.9	62.6 ± 15.9	<0.001
SSV score	25.3 ± 8.7	66.5 ± 20.1	<0.001
Active ROM (degree)			
FE (°)	75 ± 28	126 ± 41	0.001
ABD (°)	56 ± 15	85 ± 24	<0.001
ER at side (°)	32 ± 12	33 ± 15	0.730
IR at back^a^	0.7 ± 0.6	2.7 ± 1.4	<0.001
IR strength, mean ± SD (range)	1.0 ± 0.0	3.5 ± 0.9	<0.001
Anterior HH subluxation, *n* (%)	4 (36.3)	3 (27.2)	0.676
Proximal HH migration, *n* (%)	2 (18.1)	3 (27.2)	0.588

*Note*: Unless otherwise noted, values are mean ± standard deviation. The significant *p*‐value is below 0.05.

Abbreviations: ABD, abduction; ER, external rotation; FE, forward elevation; HH, humerus head; IR, internal rotation; ROM, range of motion; SD, standard deviation; SSV, subjective shoulder value; VAS, visual analogue scale.

^a^ Internal rotation was measured as the level that could be reached by the G‐0, S‐1, L5‐2, L3‐3, L1‐4, T12‐5, T10‐6, T8‐7, T6‐8.

## DISCUSSION

The full arthroscopic combined LDTM transfer resulted in significant improvements in pain relief and shoulder function, particularly in IR for ROM and strength. There was no significant complication and no progression of glenohumeral arthritis observed by the final follow‐up.

Recently, LD transfer has gained attention as a treatment option for irreparable subscapularis tears for its favourable outcomes [13, 24]. Also, a recent long‐term study involving 30 patients with an average follow‐up of 8.7 years documented significant improvements in pain, IR ROM, and IR strength. Notably, these benefits were maintained from short‐term to long‐term follow‐up, and the progression of cuff tear arthropathy remained low‐grade [3]. However, Kany et al. [13] noted that LD transfer alone may be insufficient to restore anterior subluxation of the glenohumeral joint. In contrast, the combined LDTM transfer may produce a more powerful posterior line of pull, thereby enhancing joint stability and rebalancing force coupling [6, 12]. Additionally, study by Boileau et al. [8] indicated that the LD tendon is often thin and prone to tearing, and showed that combining LDTM transfer resulted in more stable handling and fixation.

Combining LD and TM together offers several advantages. The synergistic action of the combined LD and TM tendons facilitates a rebalancing effect, enhanced by the TM tendon's scapulohumeral kinematics, which closely resemble those of the subscapularis tendon [6, 12, 16, 23]. Several biomechanical studies further support this concept. For instance, Halder et al. [16] observed in their analysis that an isolated LD transfer alters the tendon's function, shifting its role from depressing the humeral head to compressing the glenoid, which was associated with an increase in scapular tilt at higher abduction angles. In contrast, the TM consistently depresses the humeral head due to its scapular kinematics. Additionally, a study employing computational modelling by Mulla et al. [23] demonstrated that the rotator cuff tendons and the TM maintained consistent ratios across various degrees of humeral elevation. The results indicated that the LD and TM effectively function as both joint stabilisers and compressors. Furthermore, a biomechanical study by Baek et al. [6] evaluated the efficacy of combined LDTM transfers compared to LD transfers alone in a cadaveric model for irreparable subscapularis tears. The findings demonstrated that combined LDTM transfers significantly reduced superior and anteroinferior translations, as well as subacromial contact pressures across all tested conditions. In contrast, isolated LD transfers only significantly reduced superior translation during specific movements and decreased subacromial pressure under limited conditions. Nonetheless, our results suggested that preoperative anterior humeral head subluxation and proximal humeral head migration did not change significantly postoperatively. This may be attributed to posterior capsular stiffness, as fixed humeral subluxation or migration may not improve with the procedure. However, this finding is clinically insignificant, as the combined LDTM transfer resulted in improved patient‐reported outcome measures, with no cases of arthritis progression and no significant complications observed by the final follow‐up.

To date, there is limited clinical data on the outcomes of combined LDTM transfers for irreparable subscapularis tear. Baek et al. [1] demonstrated promising results for combined LDTM transfers in patients with anterosuperior irreparable rotator cuff tears. They observed notable postoperative enhancements in the mean Constant score, American Shoulder and Elbow Surgeons (ASES) score, University of California‐Los Angeles (UCLA) score, and Activities of Daily Living Index Rating (ADLIR) scores, all with *p* < 0.001, over a mean follow‐up period of 38 months. Significant improvements in active ROM for FE and IR were also noted, with *p* < 0.001. Our study aligns with the findings of Baek et al. [1], as we observed a significant reduction in pain, with the VAS score decreasing from 8.1 ± 0.9 to 2.1 ± 2.1, *p* < 0.001. Patient‐reported outcomes including Constant Score and SSV also showed marked improvements. Furthermore, significant gains in ROM were observed for FE, ABD and IR (*p* < 0.001), while IR strength improved from 1.0 ± 0.0 to 3.5 ± 0.9 (*p* < 0.001). Our study reported a low retear rate, consistent with the findings of Baek et al. [1]. This may be attributed to the anatomical characteristics of the combined LDTM muscles. As highlighted by Boileau et al. [8], the combined LDTM transfer offers a thicker and stronger construct compared to the thin tendinous portion of the LD tendon alone, which potentially reduces the risk of retear. Additionally, our study demonstrated no progression of arthritis, which may be attributed to the biomechanical properties and scapular kinematics provided by the TM muscle, as noted in the aforementioned biomechanical studies [6, 12, 16, 23]. However, when incorporating the TM muscle into the transfer, there is a theoretical risk that the axillary nerve may come into contact with the TM as it traverses the quadrilateral space [12]. Increased tension and kinking of the nerve could potentially occur following the transfer of the TM to the upper portion of the subscapularis insertion. Baek et al. [1] previously reported one case of transient axillary nerve impingement that resolved conservatively. In our study, no axillary nerve impingement was observed, likely due to the more distal reinsertion of the LDTM onto the lesser tuberosity compared to the cadaveric technique described by Elhassan et al. [12].

Although this is the first study to evaluate full arthroscopic LDTM transfer for irreparable subscapularis tears, it has several limitations. First, the relatively small number of patients, due to the low incidence of irreparable subscapularis tears, increases the risk of a Type II error. Second, the short follow‐up duration of at least 24 months leaves the long‐term effectiveness of the procedure uncertain and necessitates future study. Additionally, potential biases from concomitant procedures—such as long head of the biceps tenotomy/tenodesis and supraspinatus tendon repair—may have influenced the outcomes. Furthermore, there is a potential bias regarding the influence of irreparable supraspinatus tears associated with subscapularis tears, as no additional surgical procedures were performed for patients with concomitant irreparable supraspinatus tears. Use of different imaging modalities for preoperative and postoperative assessments may introduce variability in the detection of tendon integrity and retears. Lastly, we did not compare our cohort with patients who underwent alternative techniques, such as LD tendon transfer alone, PM tendon transfer, ACR, or reverse total shoulder arthroplasty, which limits our ability to determine the most viable treatment option.

## CONCLUSION

Full arthroscopic combined LDTM transfer demonstrates promising clinical and radiological short‐term outcomes for patients with irreparable subscapularis tears. The procedure resulted in substantial improvements in pain relief and functional outcomes, particularly in IR for both ROM and strength. Importantly, no significant complications or progression of glenohumeral arthritis were observed by the final follow‐up.

## AUTHOR CONTRIBUTIONS

**Bo Taek Kim**: Conceptualization; methology; data curation; formal analysis; writing—original draft; validation; visualisation. **Luis Alfredo Miranda**: Conceptualization; data curation; formal analysis. **Chang Hee Baek**: Conceptualization; methology; data curation. **Jung Gon Kim**: Methology; data curation; formal analysis. **Luis Leoncio Temoche Diaz**: Methology; formal analysis. **Gyu Rim Baek**: Data curation; formal analysis. **Jean Kany**: Conceptualization; methology; data curation; formal analysis; validation; supervision.

## CONFLICT OF INTEREST STATEMENT

The authors declare no conflicts of interest.

## ETHICS STATEMENT

This study was Approved by the Institutional Review Board. This study was conducted in accordance with the Code of Ethics of the World Medical Association (Declaration of Helsinki). The authors collectively declare that the requirement for informed consent was waived owing to the retrospective design of the study and the lack of additional harm to the patients.

## Supporting information

ICMJE combined.

## Data Availability

Data available on request due to privacy/ethical restrictions.
